# On interrogating electron microscopy images to discover proteins in the cell

**DOI:** 10.1107/S2052252525001861

**Published:** 2025-03-03

**Authors:** Jose-Maria Carazo

**Affiliations:** a Spanish National Center for Biotechnology, CNB-CSIC Darwin 3 28049Madrid Spain

**Keywords:** cryo-EM, cryo-electron microscopy, protein structure, template matching, image processing

## Abstract

Interrogating individual two-dimensional (2D) cryo-EM images for the presence of defined three-dimensional (3D) structures that correspond to previously known (or predicted) macromolecular complexes is very challenging, but offers attractive opportunities for the analysis of large numbers of specimens. The work of Zhang *et al.*[(2025), *IUCrJ*, **12**, 155–176] represents a significant step forward towards this goal.

Many times the difference between success and failure lies in the precise way in which we formulate the question that we are interested in, and the work of Zhang *et al.* (2025 ▸) reported in this issue of *IUCrJ* is an excellent example of this situation. They make full use of structural biology data, in the form of cryogenic electron microscopy (cryo-EM) images, with the goal of totally *de novo* biological discovery, a new frontier for structural biology (Schwalbe *et al.*, 2024 ▸). The samples they work with correspond either to cells, sections of cells or complex mixtures; they do not know *a priori* the molecular identity of the macromolecules they are observing. They solve the puzzle, and this is the main conceptual innovation of this work, by interrogating individual two-dimensional (2D) images for the presence of defined three-dimensional (3D) structures that correspond to previously known macromolecular complexes; interrogating structures are referred to as ‘templates’ and the query operation as ‘template matching’ – more precisely in this case, ‘2D template matching (2DTM)’ (Fig. 1 ▸).

The number of experimentally solved structures in the Protein Data Bank (Berman *et al.*, 2000 ▸) is very large, but still finite in number and somewhat restricted in diversity. In fact, the scarcity of data, together with the relative simplicity of the first generation of ‘interrogating’ tools, were the main drawbacks of the authors’ first approaches to this topic (Rickgauer *et al.*, 2017 ▸). As for the latter, the current work of Zhang *et al.* presents a much improved statistical approach solving many of the issues of previous methods, and for the former, the eruption of accurate prediction tools, like AlphaFold (Jumper *et al.* 2021 ▸), has completely changed the landscape. Thanks to these structure prediction capabilities, genomics, proteomics and structural biology have all converged into truly spatial discovery tools in the context of the cell.

Zhang *et al.* (2025 ▸), together with their previous work (Rickgauer *et al.*, 2017 ▸), also make a remarkable technical innovation: They choose to work with 2D images, rather than 3D volumes, to detect individual proteins, even if these 2D images are known to be very complex to interpret, since they correspond to the projection, to the ‘collapse’, of the whole 3D volume into just one plane (a plane parallel to the cryo-EM grid, perpendicular to the electron beam, with no tilt). Information superposition makes the interpretation of these images very challenging. The reason for even attempting this challenge is technical simplicity, connected to the potential for high-throughput data analysis. Indeed, anything in 3D is far more complex than in 2D, starting from the collection of data for a tilt series instead of just one image, and followed by the processing workflows. On the positive side, concentrating the total electron dose in just one image allows one to increase the signal-to-noise ratio (SNR) in that image by roughly an order of magnitude with respect to any of the individual tilt images, a very significant factor for any signal processing operation. Still, information superposition in any one image of a complex cellular environment is very significant – so significant as to cast doubts over the very feasibility of the whole approach, specially when the SNR is low, as is typically the case in cryo-EM. However, the reality is that 2D template matching works [as reviewed by Lucas (2023 ▸)], although perhaps not for all specimens. Indeed, the original formulation presented some limitations, partly due to statistical shortcomings in the 2DTM data analysis, that resulted in the applicability being dependent on the size and shape of the possible interrogating structures; many of these shortcomings have been thoroughly addressed by Zhang *et al.*

Another aspect of the work of Zhang *et al.* is that they explore a blurred line between working with 2D untilted images, a procedure typical for the task of structure determination of purified macromolecular samples (normally referred to as ‘single particle analysis’), and working with complete tilt series of micrographs to combine them and obtaining a volume, a 3D signal, in which there is no longer any cellular feature superposition and the cellular environment can be directly interrogated (an approach referred to as ‘tomography’). Indeed, the concept of using (3D) ‘templates’ in cryo-EM appeared early in tomography (Böhm *et al.*, 2000 ▸), although the combination of requiring high-resolution tomograms and accurate structural prediction tools delayed the best-known success stories to only recent years (Cruz-León *et al.*, 2024 ▸; Xue *et al.*, 2022 ▸). Zhang *et al.*’s innovative 2D template matching approach completely bypasses the need for complex tomographic data collection and processing, at the cost of dealing with intrinsically complex images that might not provide sufficient information for protein identification in all cases, but will certainly work in an increasing number of cases as 2D interrogation tools continue to be developed.

Surely we are not at the end of the road regarding 2D template matching developments and 2D interrogation in general, since the opportunities offered by this approach make it very attractive in terms of analysing large numbers of specimens. Zhang *et al.* present a significant step further, working on the statistical characterization of the interrogating tools; the future, perhaps the near future, may see further improvements using artificial intelligence approaches, including elaborate simulations of the cellular environment. The avenue to spatial biological discovery in the cell is a reality, the door is open to innovations in image interrogation, the future is tantalizing.

## Figures and Tables

**Figure 1:**
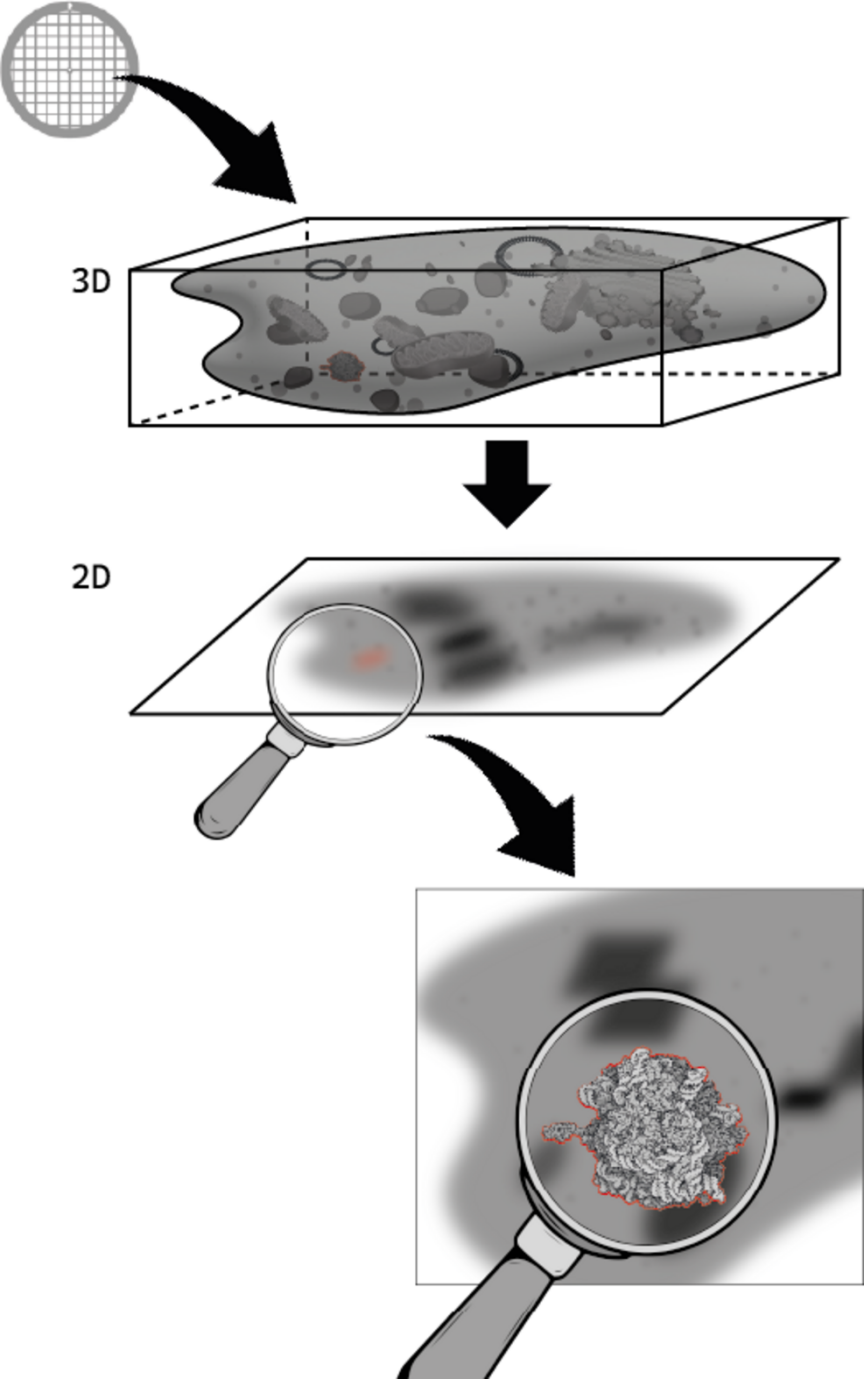
The application of 2D template matching (2DTM) starts from a very blurred cryo-EM image that corresponds to the 2D projection of a complex 3D cellular environment. In this figure, that corresponds to a conceptual workflow from top to bottom. A cryo-EM grid is shown at the top, on which a certain sample is deposited (a 3D object). An image is then acquired at the microscope, corresponding to a 2D projection of the 3D sample, resulting in a blurred image. 2DTM is then represented by the magnifying glass, whose goal is to detect the presence of a certain protein in the blurred image. This identification task is achieved (at the bottom) thanks to our knowledge of the three-dimensional structure of the macromolecular complex under investigation, as if the magnifying glass was comparing the known (or predicted) 3D structure with the experimental 2D image.
